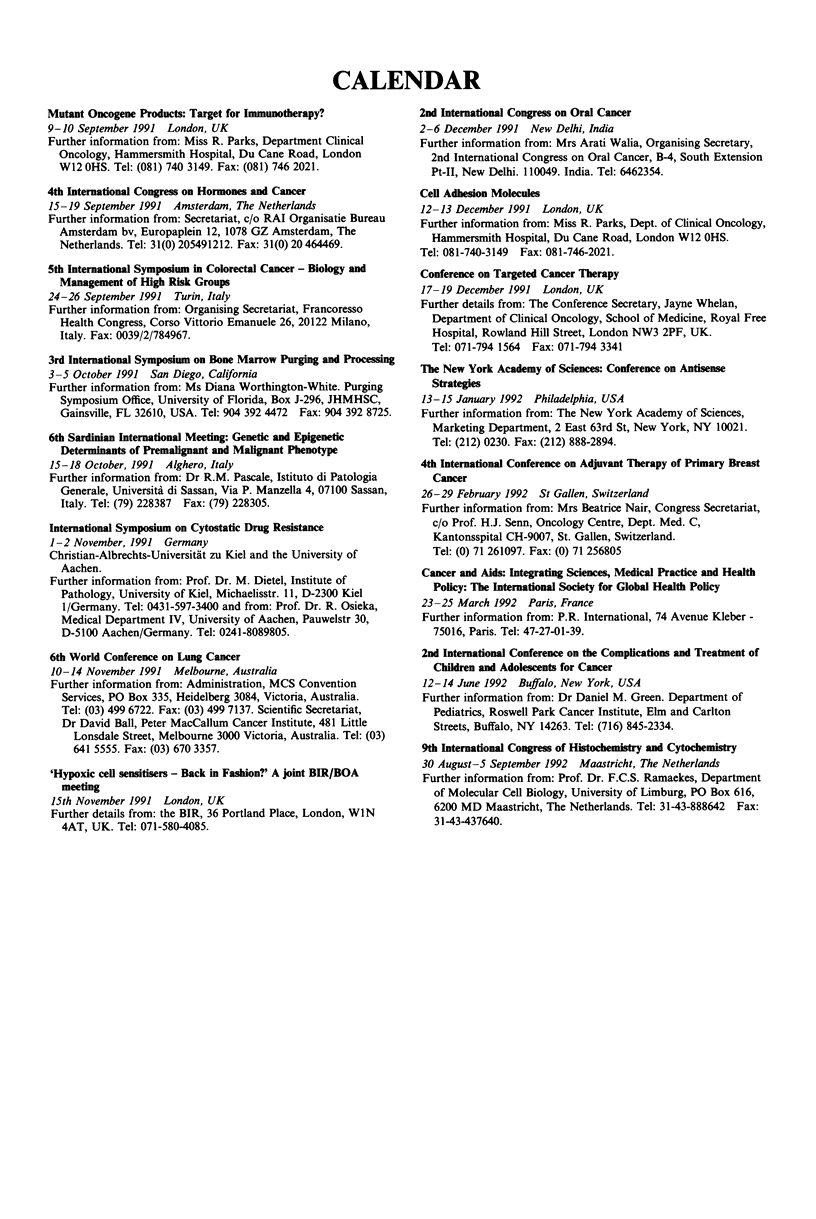# Calendar

**Published:** 1991-08

**Authors:** 


### CALENDAR

**Mutant Oncogene Products: Target for Immunotherapy?**
*9–10 September 1991 London, UK*
Further information from: Miss R. Parks, Department Clinical Oncology, Hammersmith Hospital, Du Cane Road, London W12 0HS. Tel: (081) 740 3149. Fax: (081) 746 2021.

**4th International Congress on Hormones and Cancer**
*15–19 September 1991 Amsterdam, The Netherlands*
Further information from: Secretariat, c/o RAI Organisatie Bureau Amsterdam bv, Europaplein 12, 1078 GZ Amsterdam, The Netherlands. Tel: 31(0) 205491212. Fax: 31(0) 20 464469.

**5th International Symposium in Colorectal Cancer – Biology and Management of High Risk Groups**
*24–26 September 1991 Turin, Italy*
Further information from: Organising Secretariat, Francoresso Health Congress, Corso Vittorio Emanuele 26, 20122 Milano, Italy. Fax: 0039/2/784967.

**3rd International Symposium on Bone Marrow Purging and Processing**
*3–5 October 1991 San Diego, California*
Further information from: Ms Diana Worthington-White. Purging Symposium Office, University of Florida, Box J-296, JHMHSC, Gainsville, FL 32610, USA. Tel: 904 392 4472 Fax: 904 392 8725.

**6th Sardinian International Meeting: Genetic and Epigenetic Determinants of Premalignant and Malignant Phenotype**
*15–18 October, 1991 Alghero, Italy*
Further information from: Dr R.M. Pascale, Istituto di Patologia Generale, Università di Sassan, Via P. Manzella 4, 07100 Sassan, Italy. Tel: (79) 228387 Fax: (79) 228305.

**International Symposium on Cytostatic Drug Resistance**
*1–2 November, 1991 Germany*
Christian-Albrechts-Universität zu Kiel and the University of Aachen.
Further information from: Prof. Dr. M. Dietel, Institute of Pathology, University of Kiel, Michaelisstr. 11, D-2300 Kiel 1/Germany. Tel: 0431-597-3400 and from: Prof. Dr. R. Osieka, Medical Department IV, University of Aachen, Pauwelstr 30, D-5100 Aachen/Germany. Tel: 0241-8089805.

**6th World Conference on Lung Cancer**
*10–14 November 1991 Melbourne, Australia*
Further information from: Administration, MCS Convention Services, PO Box 335, Heidelberg 3084, Victoria, Australia. Tel: (03) 499 6722. Fax: (03) 499 7137. Scientific Secretariat, Dr David Ball, Peter MacCallum Cancer Institute, 481 Little Lonsdale Street, Melbourne 3000 Victoria, Australia. Tel: (03) 641 5555. Fax: (03) 670 3357.

**'Hypoxic cell sensitisers – Back in Fashion?' A joint BIR/BOA meeting**
*15th November 1991 London, UK*
Further details from: the BIR, 36 Portland Place, London, W1N 4AT, UK. Tel: 071-580-4085.

**2nd International Congress on Oral Cancer**
*2–6 December 1991 New Delhi, India*
Further information from: Mrs Arati Walia, Organising Secretary, 2nd International Congress on Oral Cancer, B-4, South Extension Pt-II, New Delhi. 110049. India. Tel: 6462354.

**Cell Adhesion Molecules**
*12–13 December 1991 London, UK*
Further information from: Miss R. Parks, Dept. of Clinical Oncology, Hammersmith Hospital, Du Cane Road, London W12 0HS.
Tel: 081-740-3149 Fax: 081-746-2021.

**Conference on Targeted Cancer Therapy**
*17–19 December 1991 London, UK*
Further details from: The Conference Secretary, Jayne Whelan, Department of Clinical Oncology, School of Medicine, Royal Free Hospital, Rowland Hill Street, London NW3 2PF, UK. Tel: 071-794 1564 Fax: 071-794 3341

**The New York Academy of Sciences: Conference on Antisense Strategies**
*13–15 January 1992 Philadelphia, USA*
Further information from: The New York Academy of Sciences, Marketing Department, 2 East 63rd St, New York, NY 10021. Tel: (212) 0230. Fax: (212) 888-2894.

**4th International Conference on Adjuvant Therapy of Primary Breast Cancer**
*26–29 February 1992 St Gallen, Switzerland*
Further information from: Mrs Beatrice Nair, Congress Secretariat, c/o Prof. H.J. Senn, Oncology Centre, Dept. Med. C, Kantonsspital CH-9007, St. Gallen, Switzerland. Tel: (0) 71 261097. Fax: (0) 71 256805

**Cancer and Aids: Integrating Sciences, Medical Practice and Health Policy: The International Society for Global Health Policy**
*23–25 March 1992 Paris, France*
Further information from: P.R. International, 74 Avenue Kleber - 75016, Paris. Tel: 47-27-01-39.

**2nd International Conference on the Complications and Treatment of Children and Adolescents for Cancer**
*12–14 June 1992 Buffalo, New York, USA*
Further information from: Dr Daniel M. Green. Department of Pediatrics, Roswell Park Cancer Institute, Elm and Carlton Streets, Buffalo, NY 14263. Tel: (716) 845-2334.

**9th International Congress of Histochemistry and Cytochemistry**
*30 August–5 September 1992 Maastricht, The Netherlands*
Further information from: Prof. Dr. F.C.S. Ramaekes, Department of Molecular Cell Biology, University of Limburg, PO Box 616, 6200 MD Maastricht, The Netherlands. Tel: 31-43-888642 Fax: 31-43-437640.